# Depression and anxiety prevalence and correlations among cancer patients at Tikur Anbesa Hospital in Addis Ababa, Ethiopia, 2018: Cross-sectional study

**DOI:** 10.3389/fpsyt.2022.939043

**Published:** 2022-09-23

**Authors:** Yacob Abraham, Meseret G/Tsadik, Abebaw Gebeyehu, Tolesa Fanta, Tebeje Ashegu

**Affiliations:** ^1^College of Medicine and Health Sciences, Hawassa University, Hawassa, Ethiopia; ^2^Research and Training directorate, Amanuel Mental Specialized Hospital, Addis Ababa, Ethiopia; ^3^College of Medicine and Health Sciences, Institute of Public Health, University of Gondar, Gondar, Ethiopia

**Keywords:** depression, anxiety, cancer, patients, Addis Ababa, Ethiopia

## Abstract

**Background:**

People with cancer, as well as their family members and loved ones, frequently experience distress. Distress can sometimes escalate from a normal level to one that interferes with therapy, makes it difficult for you to function or cope, and has an impact on many aspects life. About 1 in 4 people with cancer experience major or clinical depression, and anxiety is also a common problem for cancer patients. Anxiety and depression are the most familiar mental illnesses among cancer patients.

**Objectives:**

The objectives of this study was to assess the prevalence and correlates of depression and anxiety among cancer patients attending treatment at Tikur Anbessa specialized hospital.

**Methods:**

Hospital based cross-sectional study was conducted from 15 April to 15 May 2018. This study was conducted at Tikur Anbessa Specialized Hospital is in Addis Ababa, capital city of Ethiopia. A pretested interviewer administered questionnaire was used to collect data by trained psychiatry nurses. The Hospital Anxiety and Depression Scale was used to collect an outcome variable (the presence of anxiety and depression).

**Result:**

According to the study, anxiety and depression were present in 54.6 percent and 40.4 percent of the Tikur Anbessa Specialized Hospital's cancer patients, respectively. Factors that were strongly associated with depression were being a woman, having less education, bleeding right now, being younger (30 to 39 years old), and suffering discomfort. With AOR of 2.18 (1.38–3.44), 1.73 (1.10–2.85), 2.57 (1.61–4.11), 2.28 (1.12–4.63), 1.64 (1.00–2.69), respectively with 95% CI. On the other hand factors significantly associated with anxiety among cancer patients attending treatment at Tikur Anbessa specialized hospital were marital status with AOR 2.10 (1.01–4.02), feeling discomfort 2.06 (1.00–3.03), and bleeding 3.52 (2.31–5.64.

**Conclusion:**

Guidelines for screening and treating depression and anxiety in cancer patients should be developed by psychiatry departments in collaboration with oncology department. Oncology and psychiatry department better work and capacitate link to help for good of patients. To enhance and widen the current findings, additional research on depression and anxiety risk factors should be done.

## Introduction

People with cancer, as well as their family members and loved ones, frequently experience distress. Distress can sometimes escalate from a normal level to one that interferes with therapy, makes it difficult for you to function or cope, and has an impact on many aspects life (1). About 1 in 4 people with cancer experience major or clinical depression, and anxiety is also a common problem for cancer patients (2). Anxiety and depression are the most familiar mental illnesses among cancer patients (3).

According to a cross-sectional study conducted in Pakistan, 66 percent of cancer patients suffer from depression and anxiety (4). In Thailand, researchers discovered that having a higher pain score, receiving less therapy, getting older, and being female were all linked to a higher likelihood of depression (5).

A prospective, multicenter cohort done in Spanish hospital reported that prevalence of anxiety and depression was 49.8 and 36.6%, respectively. Women and younger individuals were more anxious and depressed than men and seniors (6).

In a study conducted in Nigeria, depression was found to be prevalent in 40.3 percent of the population; not being married, having poor social support, and having advanced cancer stage were all found to be significantly associated with depression (7). According to a study done in Greece, women with low levels of education are more likely to continue to feel depressed and anxious (8). According to an American meta-analysis study, depressed patients were less likely than non-depressed patients to take their medication as prescribed (9).

According to one study, the occurrence of depression in chronic disease patients was linked to a poor treatment outcome (10). The high cost and domination of psychological distress among cancer patients, as well as the impairment it causes and the diagnostic and therapeutic uncertainties surrounding it, make it a priority for research. The goal of this study was to determine the prevalence of depression and anxiety in cancer patients, as well as the associated factors associated with anxiety and depression which escort cancer patients to suffer more, resulting in poor quality of life and premature death.

## Methods and materials

### Study design and period

Hospital based cross-sectional study was conducted from 15 April to 15 May 2018.

### Study setting

This study was conducted at Tikur Anbessa Specialized Hospital is in Addis Ababa, capital city of Ethiopia. This Hospital is giving health services of medical management, surgical intervention, obstetric and gynecological management, pediatric and other essential services for different population, the Hospital has oncology department which serves for patients from different regions of the country.

### Source population

All cancer patients attending oncology clinic at Tikur Anbessa Specialized Hospital.

### Study population

All cancer patients attending oncology clinic at Tikur Anbessa Specialized Hospital during study period.

### Inclusion and exclusion criteria

#### Inclusion criteria

The sample includes participant who fulfills age 18 and above, tested positive for cancer and currently on treatment at Tikur Anbessa Specialized Hospital.

#### Exclusion criteria

Those who are unable to communicate due to illness.

### Sample size determination and sampling procedures

#### Sample size determination

The maximum number of sample required for this study was determined by using single population proportion formula considering the following assumptions:


$$n=\frac{\left(Z\alpha 2{)}^{2}\;\right)P(1-P)}{d^{2}}$$

*n* = minimum sample size required for the study.

*Z* = standard normal distribution with confidence interval of 95%, *Z* = 1.96.

*d* = Absolute precision or tolerable margin of error. *d* = 0.05.

*P* = is the anticipated population proportion.

Since no similar study conducted previously in Ethiopia, we use the prevalence of anxiety and depression disorder among cancer patients 50%. Therefore, 50% was used to anticipate the proportion of the population of cancer patients who experience anxiety and depression disorder.

Therefore $n\;=\;\frac{0.5\left(1-0.5\right)1.96^{2}}{{\left(0.05\right)}^{2}}$ = 384

Adding 10% non-response rate, the final sample size was **423**.

#### Sampling technique

Systematic random sampling technique was used to select participants.

### Operational definitions

1. **A possible presence of anxiety** in this study is defined as a cut-off point ≥8 (>8) during HADS A-7 Screening.
2. **A possible presence of depression** in this study is defined as a cut-off point ≥8 (>8) during HADS D-7 Screening.

#### Data quality control issues

Ensured by conducting the pretest among 5% sample. Training was given for the data collectors on the data collection tool and sampling techniques and procedures. Supervision was held regularly and necessary feedback was offered to data collectors.

#### Data collection

A pretested interviewer administered questionnaire was used to collect data by trained psychiatry nurses. The data collection equipment was made up of different parts. The first section contains socio-demographic information (age, education, occupation, marital status and others). The Hospital Anxiety and Depression Scale was used to collect an outcome variable (the presence of anxiety and depression) (HADS). The HADS is a 14-item questionnaire that is often used to screen for anxiety and depressive symptoms. For anxiety and depression, the 14-item scale was divided into two 7-item subscales. It was validated in Ethiopia and found to have an internal consistency of 0.78 for anxiety subscales, 0.76 for depression subscales, and 0.87 for the entire scale. A cutoff score of more than or equal to 8 is used on the measures for anxiety and depression (11).

#### Data processing and analysis

The data was entered into Epi Info version 7 and then exported to SPSS version 20 for analysis. The presence of statistically significant connections between the independent and dependent variables was determined using bivariate and multivariable analysis. Multivariable analyses with a *P*-value of 0.05 were considered statistically significant.

### Ethical consideration

Ethical clearance was obtained from Ethical Review Board of University of Gondar and Amanuel mental specialized hospital. Consent was obtained from each study participant after the study was explained to them in detail by the data collectors. They also informed that they could quit at any time.

## Result

The total number of participants was 423, with a 100 percent response rate. Females made up 63.8 percent of the participants, while males made up 35 percent. The participants' ages range from 30 to 80 years old, with a mean of 44.7 years and a standard deviation of 13.6 years. 54.4 percent of the total participants in the study were married, while 22.2 percent were single. Table 1 shows the socio-demographic characteristics of the study participants.

**Table 1:** Frequency distributions of socio-demographic characteristics of the respondents at TASH in Addis Ababa, Ethiopia, 2018.

**Variables**	**Frequency**	**Percentage (%)**
**Age group of the respondents**		
<30	46	10.9
30–39	111	26.2
40–49	114	27.0
50–59	82	19.4
60 and above	70	
**Sex**		
Male	149	35.2
Female	274	64.8
**Religion**		
Orthodox	288	68.1
Muslim	75	17.7
Protestant	59	13.9
Other	1	0.2
**Marital status**		
Single	94	22.2
Married	230	54.4
Separated	27	6.4
Divorced	23	5.4
Widowed	49	11.6
**Occupational status**		
Government employed	86	20.3
Merchants	33	7.8
Farmer	130	27.7
Student	23	5.4
Daily laborer	42	7.6
House wife	80	18.9
Job less	29	2.1
**Educational status**		
Not formally educated	176	41.6
Read and write	6	1.4
Primary school	68	16.1
Secondary school	108	25.5
College /university	65	15.4
**Ethnicity**		
Amhara	182	43.0
Oromo	113	26.7
Tigre	44	10.4
Gurage	64	15.1
Other*	20	4.7
**Living status**		
With family	325	74.9
Alone	98	20.8

### Prevalence of depression

To determine presence of depression and anxiety test scored done, a value of 0 to 3 is assigned for each answer and then total score compared. Final participants were asked to rate how they have been feeling for the last 2 weeks. The minimum and maximum score were 0 to 21.

**Figure 1:**
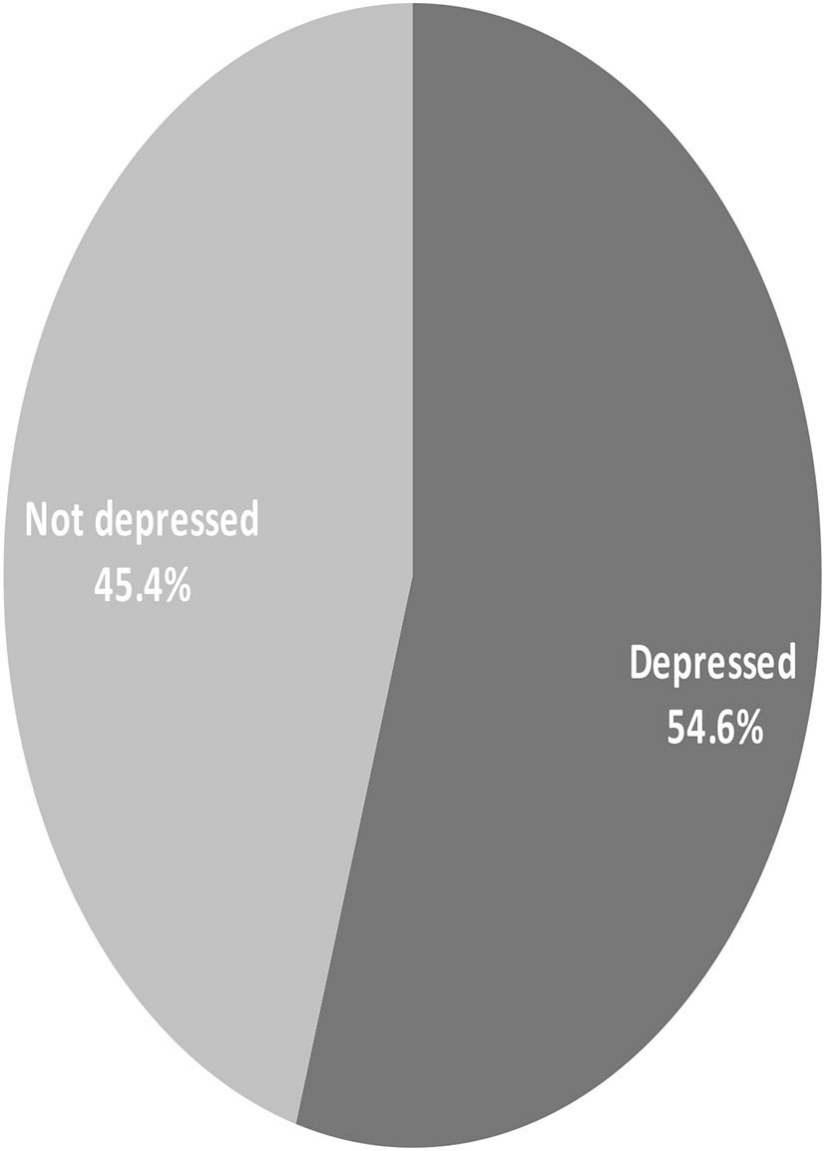
Magnitude of depression among cancer patient participants, at TASH in Addis Ababa Ethiopia, 2018.

Hospital anxiety depression scale (HADS) were interpreted for participants who took the test and got a total score of 8 or higher were indicative of depression, accordingly the study showed the prevalence of depressed cancer patients 54.6% (Figure 1).

### Prevalence of anxiety

**Figure 2:**
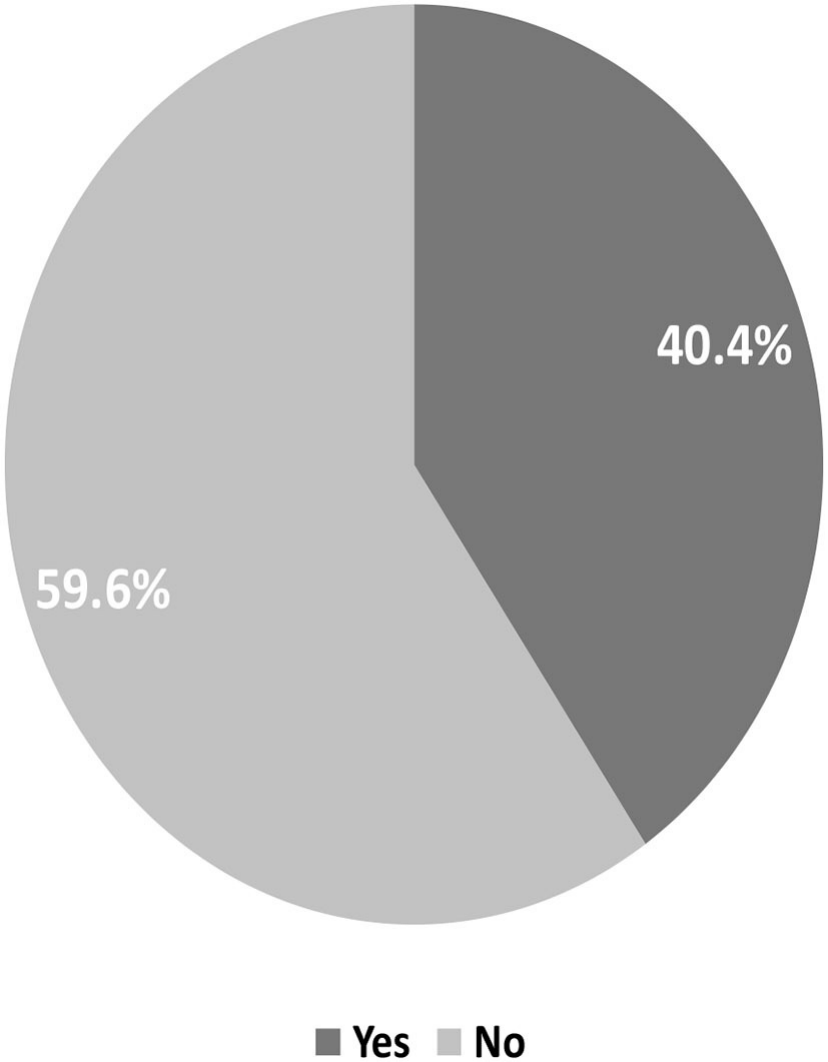
Magnitude of anxiety among cancer patients' participants at TASH in Addis Ababa Ethiopia, 2018.

Hospital anxiety depression scale (HADS) were interpreted for participants who took the test and got a total score of 8 or higher were indicative of anxiety, with cut-off point 8, the prevalence of anxiety among cancer patients was 40.4% (Figure 2).

### Factors associated with depression

According to the result of this study; Gender, educational status, current bleeding, age groups (30 to 39) years, and feeling pain had association before and after adjustment as illustrated in Table 2. With AOR of 2.18 (1.38–3.44), 1.73 (1.10–2.85), 2.57 (1.61–4.11), 2.28 (1.12–4.63), 1.64 (1.00–2.69), respectively show significant association with 95% Cl. Females were 2.18 times more likely than males to acquire depression, as indicated by the adjusted odd ratio, and persons who experienced bleeding from a cancer site were 2.18 times more likely to develop depression. When there was bleeding from a cancer site, patients were 2.57 times more likely to develop depression. In comparison to those aged 60 and up, those aged 30 to 39 were 2.28 times more likely to acquire depression. Patients with pain were 1.64 times more likely to become depressed than those without pain. Participants who couldn't read or write were 1.73 times more likely to acquire depression than those who had a diploma or higher.

**Table 2:** Bivariate and Multivariate logistic analysis result of depression study subjects among adult patients attending Oncology clinic at Tikur Anbessa Hospital, Addis Ababa, Ethiopia, 2018.

**Variables**	**Depression**		**Crude OR ( 95% CI)**	**Adjusted OR (95% CI)**
	**Yes (%)**	**No (%)**		
**Gender**				
Male	66 (28.6)	83 (43.2)	**1.00**	**1.00**
Female	165 (71.4)	109 (56.8)	2.41 (1.49–3.89)^*^	2.18 (1.38–3.44)^*^
**Age groups**				
<30	22 (9.5)	24 (12.5)	3.15 (1.20–8.32)^*^	2.16 (0.92–6.06)
30–39	52 (22.5)	59 (30.7)	3.74 (1.29–5.84)^*^	2.28 (1.12–4.63)^*^
40–49	63 (27.3)	51 (26.6)	1.60 (0.81–3.16)	1.50 (0.76–2.93)
50–59	56 (24.2)	26 (13.5)	0.74 (0.37–1.50)	0.74 (0.36–1.48)
60+	38 (16.5)	32 (16.7)	1.00	1.00
**Marital status**				
Single	49 (21.2)	45 (23.4)	0.51 (0.24–1.08)	0.51 (0.24–1.09)
Married	125 (54.1)	105 (54.7)	0.84 (0.46–1.51)	0.77 (0.46–1.30)
Divorced	57 (24.7)	42 (21.9)	1.00	1.00
**Educational status**				
Not formally educated	102 (44.2)	80 (41.7)	1.70 (1.03–2.80)^*^	1.73 (1.10–2.85)^*^
Literate	129 (55.8)	112 (58.3)	1.00	1.00
**Living status**				
With family	175 (75.8)	142 (74.0)	0.83 (0.46–1.49)	0.83 (0.46–2.00)
Alone and other	56 (24.2)	50 (26.0)	1.00	1.00
**Bleeding for current illness**				
Yes	91 (39.4)	35 (18.2)	**2.92 (1.81–4.70)**	**2.57 (1.61–4.11)**
No	140 (60.6)	157 (81.8)		
**Feeling pain**
Yes	195 (84.4)	137 (71.4)	**2.17 (1.32–3.59)**	**1.64 (1.00–2.69)**
No	36 (15.6)	55 (28.6)	
**Limitations of general job activity**				
Yes	212 (91.8)	170 (89.0)	1.76 (0.76–4.10)	1.80 (0.78–4.15)
No	19 (8.2)	21 (11.0)		
**Swelling on a body**				
Yes	158 (68.4)	117 (60.9)	1.13 (0.70–1.85)	1.13 (0.70–1.85)
No	73 (31.6)	75 (39.1)		
**Alcohol use**				
Yes	15 (6.5)	12 (6.2)	0.91 (0.39–2.10)	0.91 (0.39–2.10)
No	216 (93.5)	180 (93.8)		
**Khat use**				
Yes	15 (6.5)	14 (7.3)	1.17 (0.52–2.62)	1.13 (0.53–2.41)
No	216 (93.5)	178 (92.7)		

(n = 423).

* Shows p < 0.05, and bold values shows significant association.

### Factors associated with anxiety

According to the result of this study marital status, bleeding, feeling pain show association before and after adjustment as shown in Table 3.

**Table 3:** Bivariate and Multivariate logistic analysis result of Anxiety study subjects among adults patients attending Oncology clinic at Tikur AnbessaHospital, Addis Ababa, Ethiopia, 2018.

**Variables**	**Anxiety**		**Crud OR 95% CI**	**Adjusted OR**
	**Yes (%)**	**No (%)**		
**Marital status**				
Married	89 (52.0)	141 (56.0)	1.00	1.00
Single	58 (33.9)	36 (14.3)	2.0 (1.0–4.4)^*^	2.10 (1.01–4.02)^*^
Divorced	24 (14.0)	75 (29.8)	051 (0.27–0.97)^*^	0.45 (0.26–1.81)
**Educational status**				
Not formally educated	57 (33.3)	125 (49.6)	1.72 (1.03–2.88)^*^‘	1.72 (1.10–1.64)
Literate	114 (66.7)	127 (50.4)	1.00	1.00
**Bleeding for current illness**				
Yes	80 (46.8)	46 (18.3)	3.51 (2.21–5.53)^*^	3.52 (2.31–5.64)^*^
No	91 (53.2)	206 (81.7)	**1.00**	**1.00**
**Feeling pain**				
Yes	162 (94.7)	170 (67.5)	1.5 (1.00–3.09)^*^	2.06 (1.00–3.03)^*^
No	9 (5.3)	82 (32.5)	**1.00**	**1.00**
**Limitations of general job activity**				
Yes	212 (91.8)	170 (89.0)	1.76 (0.76–4.10)	1.80 (0.78–4.15)
No	19 (8.2)	21 (11.0)		
**Swelling on cancer site**				
Yes	158 (68.4)	117 (60.9)	1.13 (0.70–1.85)	1.13 (0.70–1.85)
No	73 (31.6)	75 (39.1)		
**Alcohol use**				
Yes	15 (6.5)	12 (6.2)	0.91 (0.39–2.10)	0.91 (0.39–2.10)
No	216 (93.5)	180 (93.8)		
**Khatuse**				
Yes	15 (6.5)	14 (7.3)	1.17 (0.52–2.62)	1.13 (0.53–2.41)
No	216 (93.5)	178 (92.7)		

(n = 423).

* Shows p < 0.05, and also bold values shows significant association.

Marital status with AOR 2.10 (1.01–4.02), feeling discomfort 2.06 (1.00–3.03), and bleeding now 3.52 (2.31–5.64) were found to be significantly linked with 95 percent Cl.

As seen in the corrected odd ratio, single people were 2 times more likely to experience anxiety than married people and people who suffered pain. An individual who had no pain was 2 times more likely to acquire anxiety, and an individual who had bleeding from a cancer site was 3.52 times more likely to develop anxiety than an individual who had no bleeding from a cancer site.

## Discussions

The goal of this study was to find out how common anxiety and depression are among cancer patients. Using the hospital anxiety depression scale (HADS), we discovered an association between depression and common socio demographic variables, as well as an association between anxiety and socio demographic variables (HADS).

An overall prevalence of anxiety and depression was identified, with 231 (54.6%) individuals suffering from depression and 171 (40.4%) suffering from anxiety. This study's prevalence of depression (54.6%) was lower than that of a study conducted in Pakistan (66%) but greater than that of a study conducted in Nigeria (40.3%) (4, 7) and India (12) and London (13).

This study's prevalence of depression was higher than that of a similar study technique and design conducted in Babol, Iran, which was 48 percent, but prevalence of anxiety in this study was lower than that of Babol, Iran, which was 46 percent (14).

Female cancer patients were more prone to develop depressed than male cancer patients.

This conclusion is supported by findings from a Thai cancer patient study (5) and a British study (15).

Females faced added stressors of loss of physical appearance after breast surgery, which could be due to gender specific variables such as breast cancer and cervical cancer, as well as hormonal differences. The younger age group of 30 to 39 years had a significant impact on the prevalence of depression. This result is similar to that of a prior study NHS breast clinic, study report in Athens (8), and study in Pakistan (4) while the contrary is true in the study report in Iran (14).

A difference in population or culture could be the cause of the disparity.

In this study there was a statistically significant association between educational levels and depression with AOR 1.73 (1.10–2.85). The study finding reveled that patients who were not formally educated were 1.73 times more likely to develop depression than those who had a diploma or higher. This research was backed up by a study conducted in Nigeria (16). One probable explanation is that persons who are not formally educated are more likely to encounter socioeconomic stressors such as unemployment, poverty, and economic dependency, all of which might raise the chance of developing depression. People with a greater degree of education appear to use health care services more frequently than those without. Pain was independent predictor of depression in this study which is line with study done in Victoria (17).

The findings of this study revealed that marital status, pain and bleeding were all strongly linked to the prevalence of anxiety in cancer patients. Being single was 2 times more likely to acquire anxiety than being married, which is similar to a study done in Athens (8) and the link between pain and anxiety prevalence in this study was similar to a study done in Tehran (18). Research study in Nigeria reported that being unmarried associated with depression in patient with breast cancer (7).

Due to their pain, cancer patients were more likely to suffer discontent in their lives and exposed to anxiety. This finding is similar with study in Tehran (18). An individual who had bleeding from cancer site 3.52 times more likely to develop anxiety as compared to individual who had no bleeding from cancer site. One possible explanation is that people who suffer from bleeding pain have increased life dissatisfaction as a result of their sickness.

## Conclusions

Female cancer patients had a higher prevalence of depression and anxiety than male cancer patients, implying that depression and anxiety are more common in females with cancer. Being female, having a low educational status, having current bleeding, being in a younger age group, and feeling pain were all associated with a higher risk of depression, whereas being single, bleeding, and currently feeling pain were all associated with a higher risk of anxiety among cancer patients treated at Addis Ababa Tikur Anbessa Hospital in Ethiopia, 2018. Guidelines for screening and treating depression and anxiety in cancer patients should be developed by psychiatry departments in collaboration with oncology department. Oncology and psychiatry department better work and capacitate link to help for good of patients. To enhance and widen the current findings, additional research on depression and anxiety risk factors should be done.

## Data availability statement

The raw data supporting the conclusions of this article will be made available by the authors, without undue reservation.

## Ethics statement

The studies involving human participants were reviewed and approved by Ethical Review Board of University of Gondar and Amanuel mental specialized hospital. The patients/participants provided their written informed consent to participate in this study.

## Author contributions

YA: manuscript writing, analyzing, and reporting. MG and TF: report writing, analyzing, result, and discussion writing. AG: analyzing, report writing, and conclusion. TA: manuscript writing, editing result, and discussion. All authors contributed to the article and approved the submitted version.

## Funding

The study was funded in part by Amanuel Mental Specialized Hospital in Addis Ababa, Ethiopia.

## Conflict of interest

The authors declare that the research was conducted in the absence of any commercial or financial relationships that could be construed as a potential conflict of interest.

## Publisher's note

All claims expressed in this article are solely those of the authors and do not necessarily represent those of their affiliated organizations, or those of the publisher, the editors and the reviewers. Any product that may be evaluated in this article, or claim that may be made by its manufacturer, is not guaranteed or endorsed by the publisher.
